# Association of sleep-related disorders with cardiovascular disease among adults in the United States: A cross-sectional study based on national health and nutrition examination survey 2005–2008

**DOI:** 10.3389/fcvm.2022.954238

**Published:** 2022-08-04

**Authors:** Kaisaierjiang Kadier, Lian Qin, Aikeliyaer Ainiwaer, Rena Rehemuding, Diliyaer Dilixiati, Yi-Ying Du, Halimulati Maimaiti, Xiang Ma, Yi-Tong Ma

**Affiliations:** ^1^Department of Cardiology, First Affiliated Hospital of Xinjiang Medical University, Ürümqi, China; ^2^Department of Emergency Medicine, First Affiliated Hospital of Medical College, Shihezi University, Shihezi, China; ^3^Clinical Medical College, First Affiliated Hospital of Xinjiang Medical University, Ürümqi, China

**Keywords:** cardiovascular disease, congestive heart failure, coronary heart disease, sleep duration, sleep disorders, sleep-onset latency, cross-sectional study, NHANES

## Abstract

**Background and objective:**

The association between sleep-related disorders and cardiovascular diseases (CVDs) remains controversial and lacks epidemiological evidence in the general population. We investigated whether sleep-related disorders are related to CVDs in a large, nationally representative, diverse sample of American adults.

**Materials and methods:**

Data were collected from the National Health and Nutrition Examination Survey (NHANES) 2005–2008. Logistic regression was performed to explore associations of sleep-related disorders with the prevalence of total and specific CVDs. Stratified subgroup analysis was performed to exclude interactions between variables and sleep-related disorders. Non-linearity was explored using restricted cubic splines.

**Results:**

In total, 7,850 participants aged over 20 years were included. After controlling for confounders, multivariate regression analysis showed that sleep problems were associated increases in risk of 75% for CVD (OR: 1.75; 95% CI 1.41, 2.16), 128% for congestive heart failure (CHF) (OR: 2.28; 95% CI 1.69, 3.09), 44% for coronary heart disease (CHD) (OR: 1.44; 95% CI 1.12, 1.85), 96% for angina pectoris (AP) (OR: 1.96; 95% CI 1.40, 2.74), 105% for heart attack (OR: 2.05; 95% CI 1.67, 2.53) and 78% for stroke (OR: 1.78; 95% CI 1.32, 2.40). Daytime sleepiness was associated increases in risk of 54% for CVD (OR: 1.54; 95% CI 1.25, 1.89), 73% for CHF (OR: 1.73; 95% CI 1.22, 2.46), 53% for AP (OR: 1.53; 95% CI 1.12, 2.10), 51% for heart attack (OR: 1.51; 95% CI 1.18, 1.95), and 60% for stroke (OR: 1.60; 95% CI 1.09, 2.36). Participants with insufficient sleep had a 1.42-fold higher likelihood of CVD (OR: 1.42; 95% CI 1.13, 1.78) and a 1.59-fold higher likelihood of heart attack (OR: 1.59; 95% CI 1.19, 2.13) than participants with adequate sleep. Prolonged sleep-onset latency was associated with an increased risk of CVD (OR: 1.59; 95% CI 1.17, 2.15), CHF (OR: 2.08; 95% CI 1.33, 3.23) and heart attack (OR: 1.76; 95% CI 1.29, 2.41). Short sleep-onset latency was associated with a 36% reduction in stroke risk (OR: 0.64; 95% CI 0.45, 0.90). The association of sleep problems with CVD risk was more pronounced in the group younger than 60 years (p for interaction = 0.019), and the relationship between short sleep-onset latency and total CVD differed by sex (p for interaction = 0.049). Additionally, restricted cubic splines confirmed a linear relationship between sleep-onset latency time and CVD (p for non-linearity = 0.839) and a non-linear relationship between sleep duration and CVD (p for non-linearity <0.001).

**Conclusion:**

According to a limited NHANES sample used to examine sleep-related disorders and CVD, total and specific CVDs could be associated with certain sleep-related disorders. Additionally, our study uniquely indicates that CVD risk should be considered in participants younger than 60 years with sleep problems, and shortened sleep-onset latency may be a CVD protective factor in females.

## Introduction

Cardiovascular disease (CVD), including congestive heart failure (CHF), coronary heart disease (CHD), angina pectoris (AP), heart attack and stroke, is a major public health challenge and among the leading causes of mortality worldwide (1). The CVD prevalence (CHF, CHD, and stroke only) is 9.3% overall in the United States and increases with age in both males and females (2). The link between the cardiovascular system and sleep processes is bidirectional, and CVD is associated with alterations in physiological sleep and vice versa (3). In addition to well-known risk factors, research evidence suggests that multiple sleep-related disorders are important risk factors leading to CVD (4). Therefore, it is essential for health professionals and policymakers to understand the relevance of sleep-related disorders to the prevalence of CVD to develop appropriate prevention strategies as well as rational health resource allocation according to the cost burden of society.

Sleep problems and circadian rhythm disorders afflict millions of Americans (5, 6). For example, 34.8% of people do not achieve the recommended ≥7-h sleep duration (7), and approximately one-third of the adult population suffers from insomnia (8). Several studies have shown that sleep duration is associated with CVD risk and mortality, which includes CHD and stroke (9–11). However, these previous studies focused on sleep duration and did not comprehensively consider sleep behavior with an associated risk of CVD. In addition, a large, population-based study indicated that patients with different symptoms of sleep problems may carry an additional risk of up to 27 to 45% for cardiovascular events (12). However, another cohort study reported inconsistent conclusions (13). Although only a few studies have jointly assessed sleep behavior, suggesting that sleep-related disorders are associated with increased CVD risk and all-cause mortality (14–16), due to differences in population and research focus, existing research conclusions are partially contradictory and limited.

The National Health and Nutrition Examination Survey (NHANES) collected data using a complete sleep disorder questionnaire for the period 2005–2008, which is valuable for cross-sectional studies of sleep profiles in the United States. Access to a large, nationally representative United States non-institutionalized population database provides a unique opportunity to explore the associations of sleep-related disorders and the prevalence of total and specific CVDs. In addition, we also analyzed subgroups of specific populations to further study the correlations of sleep-related disorders and the prevalence of total CVD.

## Materials and methods

### Study population

This study analyzed data from individuals from the 2005 to 2008 NHANES. Conducted by the National Center for Health Statistics at the Centers for Disease Control and Prevention, NHANES is a cross-sectional, nationally representative survey of the non-institutionalized civilian population of the United States designed to examine demographic, socioeconomic, health, and nutrition information. To ensure that samples are representative, data from the NHANES are collected with a complex, multistage probability design that identifies strata based on geography and a proportion of minority populations (17).

**FIGURE 1:**
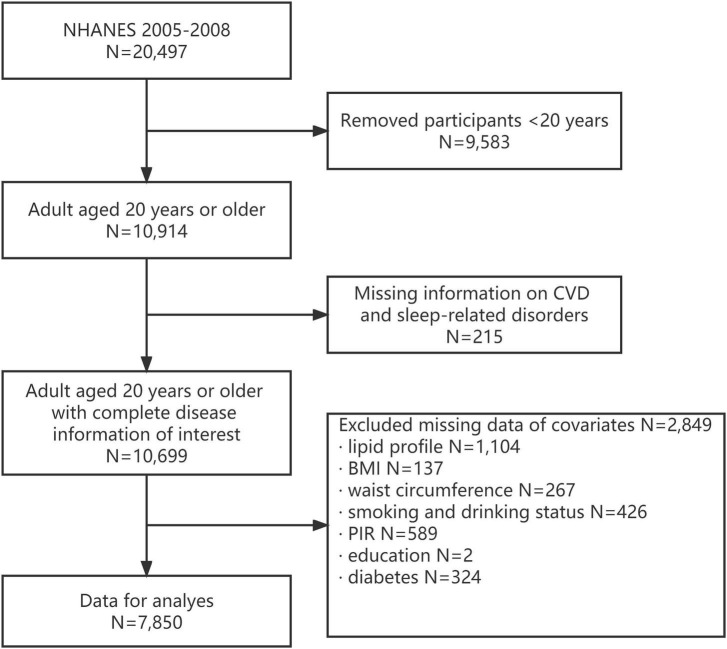
Flow chart of eligible National Health and Nutrition Examination Survey (NHANES) participants included in this study.

The 2005–2008 NHANES –are the only cycles where a complete questionnaire about sleep habits and disorders was completed. A subscale of eight questions related to general productivity from the Functional Outcomes of Sleep Questionnaire is also included (18, 19). In the 2005–2008 cycle of NHANES, 20,497 participants completed the survey. However, in the current study, individuals aged <20 years without complete information on CVDs and sleep-related disorders were excluded (*N* = 9,798). In addition, participants with missing data on covariates were excluded from the analysis (*N* = 2,849). In the end, 7,850 participants were included in the analysis (Figure 1). The NCHS Research Ethics Review Board approved the NHANES protocol, and each participant provided written informed consent. This study was designed according to the guidelines for reporting cross-sectional studies, which are specified by the Strengthening the Reporting of Observational Studies in Epidemiology (STROBE) (20).

### Assessment of cardiovascular diseases

The medical conditions section contains self-reported data from personal interviews on a wide range of health issues, including CHF, CHD, AP, heart attack, and stroke. “Has a doctor or other health professional ever told you that you have CHF/CHD/angina/heart attack/stroke?” was the question being asked to the participants. CVDs were considered to exist if any of the above questions were answered positively.

### Assessment of sleep-related disorders

In our study, we evaluated the following outcomes associated with sleep disorders: sleep duration, sleep-onset latency, obstructive sleep apnea (OSA) symptoms, sleep problems, and daytime sleepiness. From the NHANES sleep questionnaire, these results were defined as (21–24) follows.

Sleep duration: classified as insufficient (<7 h/night), normal (7–8 h/night), or excessive (>8 h/night). Sleep onset latency: categorized as short (<5 min/night), normal (5–30 min/night), or prolonged (>30 min/night).

Obstructive sleep apnea symptoms: defined as any of the following: doctor or other health professional diagnosed sleep apnea; “In the past 12 months, how often did you snore/snort, gasp, or stop breathing while you were sleeping?” answering “Occasionally (3–4 nights/week)” or “Frequently (5 or more nights/week)” to any of the questions; or feel excessively or overly sleepy during the day 16–30 times per month despite sleeping approximately 7 or more hours per night.

Sleep problems: Participants were defined as having sleep problems if they responded “Yes” to the question “Have you ever told a doctor or other health professional that you have trouble sleeping/sleep disorder; or considered frequent if self-reported” “often” or “almost always” (≥5 times/month) in response to any of the following questions: “How often have trouble falling asleep/wake up during night/wake up too early in morning?”

Daytime sleepiness: considered frequent if self-reported “often” or “almost always” (≥5 times/month) in response to any of the following questions: “In the past month, how often did you feel unrested/feel excessively or overly sleepy during the day, no matter how many hours of sleep you have?”

### Covariates

Demographic variables such as age, sex, race, education, and household poverty income ratio (PIR) were obtained during home interviews. PIR was stratified as <1.3, 1.3–1.8, and >1.8, as recorded in the original survey. Lifestyle factors such as smoking status, alcohol consumption status, and physical activity were obtained by self-reporting. Never smokers were classified as those who reported smoking less than 100 cigarettes in their life. Those who smoked more than 100 cigarettes in their life and had quit smoking were considered former smokers, and those who smoked more than 100 cigarettes in their life and smoked some days or every day were considered current smokers. Alcohol consumption status was categorized as yes or no for those who had at least 12 alcoholic drinks per year. Physical activity status was classified as vigorous, moderate or inactive according to the questionnaire. Body mass index (BMI) and waist circumference were measured at a mobile examination center using standard protocols. In the subgroup analysis, obesity was defined as a BMI ≥30, and abdominal obesity was defined as a waist circumference ≥102 cm in males and ≥88 cm in females (25). A laboratory blood analyzer was used to collect the fasting serum lipid profile, including total cholesterol (TC), HDL-cholesterol (HDL-C), and triglyceride (TG). Hypertension was defined as a diagnosis from a doctor or other health professional, average blood pressure ≥130/80 mmHg or use of medication for hypertension (26). Diabetes was defined as a diagnosis from a doctor or other health professional, HbA1c (%) >6.5, random blood glucose (mmol/l) ≥11.1, or use of medication or insulin for diabetes.

### Statistical analysis

Continuous variables are presented as the weighted mean ± standard deviation (SD) and were compared using the *T*-test, while categorical variables are presented as weighted percentages (95% confidence interval, 95% CI) and were compared using the Rao-Scott chi-square test. A multivariate logistic regression analysis was used to evaluate the correlation between sleep-related disorders and CVDs with an odds ratio (OR) and 95% CI, along with adjustment for confounding variables. Model 1 was adjusted for age, sex, and race; Model 2 was adjusted for Model 1 plus education, smoking status, alcohol consumption status, PIR, BMI, diabetes and hypertension; and Model 3 was adjusted for Model 2 plus physical activity, TC, HDL-C, and TG. Subgroup analysis stratified by age, sex, obesity, abdominal obesity, diabetes, hypertension, and physical activity was also conducted using stratified multivariate regression analysis. Additionally, the interaction test clarified the heterogeneity of correlations between subgroups. Restricted cubic splines with 3 knots, at the 10th, 50th, and 90th percentages, were used to explore the non-linear relationships of sleep duration and sleep-onset latency time with total CVD in the fully adjusted model.

The NHANES creates weights to account for the complex survey design, survey non-response, and post-stratification adjustment to match the total population living in the United States. According to the NHANES analysis guidelines (17), the study uses the 2-year MEC weight that is appropriate for the variable of interest that was collected on the smallest number of respondents, and new 4-year weights could be calculated by dividing the 2-year weights by two. All statistical analyses were performed using R software (version 4.1.3). All statistical tests were two-tailed, and a *p*-value less than 0.05 was considered statistically significant.

## Results

### Epidemiological characteristics of participants

The sample size included in the study was 7,850, representing 169,277,364 non-institutionalized adults (20 years and older) in the United States. The mean age of the population surveyed was 46.63 ± 0.45 years. Among the participants, 72.93% (62.84–83.01) were non-Hispanic white, and 50.85% (46.11–55.59) were female. Baseline characteristics were compared according to the presence or absence of CVD and are presented in Table 1. The prevalence of CVD was 7.75% (6.59, 8.91), while the prevalence rates of CHF, CHD, AP, heart attack, and stroke were 1.92% (1.63, 2.20), 3.07% (2.52, 3.63), 2.11% (1.69, 2.52), 3.12% (2.47, 3.77), and 2.69% (2.17, 3.22), respectively. Of the participants with CVD, the mean age was 64.35 ± 0.70, 55.17% (50.92, 59.43) were male and 78.22% (73.44, 83.00) were non-Hispanic white. In addition, compared with participants without CVD, there were differences in sleep duration, sleep-onset latency time, sleep problems, and OSA symptoms. Significant differences were also found in age, sex, race, education, PIR, physical activity, smoking status, alcohol consumption status, diabetes, hypertension, BMI, waist circumference, TC, HDL-C, and TGs (all *p* < 0.05).

**TABLE 1:** General characteristics of included participants (*n* = 7,850) according to the presence or absence of CVD in the NHANES 2005–2008.

Characters	Overall (*n* = 7850)	Non-CVD (*n* = 7006)	CVD (*n* = 844)	*P*-value
**Age, year**	46.63 ± 0.45	45.14 ± 0.42	64.35 ± 0.70	< 0.001
**Gender**				0.007
Male	49.15 (44.92–53.38)	48.64 (47.61–49.68)	55.17 (50.92–59.43)	
Female	50.85 (46.11–55.59)	51.36 (50.32–52.39)	44.83 (40.57–49.08)	
**Race**				0.001
Mexican American	7.75 (6.17–9.32)	8.03 (6.22–9.84)	4.40 (2.83–5.98)	
Non-Hispanic black	10.22 (8.20–12.24)	10.13 (7.57–12.69)	11.31 (8.33–14.29)	
Non-Hispanic white	72.93 (62.84–83.01)	72.48 (68.22–76.75)	78.22 (73.44–83.00)	
Other Hispanic	3.90 (2.63–5.18)	4.04 (2.70–5.37)	2.33 (0.85–3.82)	
Other race or multi-racial	5.20 (4.29–6.11)	5.32 (4.22–6.43)	3.73 (2.16–5.30)	
**Education**				< 0.001
Less than 9th grade	5.89 (4.93–6.84)	5.44 (4.54–6.34)	11.26 (8.84–13.67)	
9–11th grade	12.07 (10.02–14.13)	11.74 (9.95–13.52)	16.06 (13.47–18.65)	
High school grad	24.58 (21.65–27.50)	24.31 (22.73–25.89)	27.76 (23.12–32.41)	
College degree	30.63 (27.49–33.78)	30.94 (29.42–32.45)	27.01 (22.71–31.31)	
College or above	26.83 (23.03–30.63)	27.58 (24.48–30.68)	17.91 (13.96–21.87)	
**Poverty-income ratio**				< 0.001
<1.3	17.99 (15.85–20.13)	17.32 (15.54–19.10)	25.93 (20.92–30.93)	
1.3–1.8	9.16 (8.14–10.17)	8.88 (7.88–9.88)	12.45 (9.63–15.28)	
>1.8	72.85 (65.30–80.41)	73.80 (71.43–76.17)	61.62 (56.05–67.19)	
**Smoking status**				< 0.001
Now	23.50 (20.58–26.43)	23.75 (21.79–25.70)	20.60 (18.13–23.06)	
Former	25.17 (22.58–27.75)	23.64 (22.26–25.02)	43.34 (39.47–47.21)	
Never	51.33 (46.59–56.08)	52.62 (50.42–54.81)	36.06 (32.00–40.13)	
**Alcohol consumption status**				0.010
Yes	75.20 (67.93–82.47)	75.60 (73.28–77.91)	70.48 (65.34–75.63)	
No	24.80 (21.73–27.87)	24.40 (22.09–26.72)	29.52 (24.37–34.66)	
**Activity**				< 0.001
Vigorous	31.74 (28.29–35.20)	33.29 (30.75–35.82)	13.39 (9.69–17.09)	
Moderate	29.19 (25.97–32.41)	28.99 (26.97–31.01)	31.63 (27.02–36.25)	
Inactive	39.06 (33.93–44.20)	37.73 (34.16–41.30)	54.97 (49.88–60.07)	
**Sleep duration**				< 0.001
<7 h/night	36.34 (33.26–39.42)	35.91 (33.83–37.99)	41.43 (37.08–45.78)	
7–8 h/night	57.02 (50.95–63.09)	57.77 (55.90–59.64)	48.08 (42.80–53.36)	
>8 h/night	6.64 (5.83–7.45)	6.32 (5.62–7.01)	10.49 (7.78–13.20)	
**Sleep onset latency time**				0.001
<5 min	29.02 (25.73–32.30)	29.40 (27.75–31.06)	24.42 (19.47–29.37)	
5–30 min	53.64 (48.74–58.54)	53.84 (52.04–55.64)	51.26 (45.56–56.95)	
>30 min	17.35 (15.49–19.21)	16.76 (15.59–17.93)	24.33 (20.43–28.22)	
**Sleep problems**	41.72 (37.40–46.04)	40.51 (39.27–41.74)	56.16 (51.97–60.36)	< 0.001
**OSA symptoms**	50.82 (45.66–55.97)	50.34 (48.12–52.55)	56.52 (51.80–61.25)	0.019
**Daytime sleepiness**	29.19 (26.21–32.17)	28.92 (27.52–30.32)	32.43 (28.38–36.47)	0.106
**Hypertension**	48.40 (43.89–52.90)	45.80 (44.00–47.60)	79.31 (75.54–83.08)	< 0.001
**Diabetes**	10.05 (8.78–11.33)	8.29 (7.48–9.09)	31.07 (27.05–35.08)	< 0.001
**BMI (kg/m^2^)**	28.56 ± 0.14	28.45 ± 0.15	29.83 ± 0.23	< 0.001
**Waist circumference (cm)**	97.89 ± 0.41	97.30 ± 0.42	104.91 ± 0.55	< 0.001
**TC (mg/dL)**	198.02 ± 0.64	198.91 ± 0.70	187.31 ± 2.35	< 0.001
**HDL-cholesterol (mg/dL)**	52.94 ± 0.32	53.11 ± 0.31	50.93 ± 0.79	0.005
**TG (mg/dL)**	159.36 ± 1.94	157.96 ± 2.15	176.00 ± 7.39	0.034

Values indicate the weighted mean ± SD or weighted% (95% confidence interval). *P*-values are weighted. OSA, obstructive sleep apnea; BMI, body mass index; TC, total cholesterol; TG, triglyceride.

### Sleep-related disorders of participants

Of the total participants, 36.34% (33.26, 39.42) slept less than 7 h per night, while only 6.64% (5.83, 7.45) slept more than 8 h per night. A total of 29.02% (25.73, 32.30) of the participants reported falling asleep in 5 min or less. In contrast, 17.35% (15.49, 19.21) of participants reported taking more than 30 min to fall asleep. Moreover, 41.72% (37.40, 46.04) of participants had a combination of sleep problems, such as difficulty falling asleep, waking up during the night, or waking up too early. Daytime sleepiness and OSA symptoms were reported in 29.19% (26.21, 32.17) and 50.82% (45.66, 55.97) of participants, respectively.

### Association between sleep-related disorders and total cardiovascular disease

**FIGURE 2:**
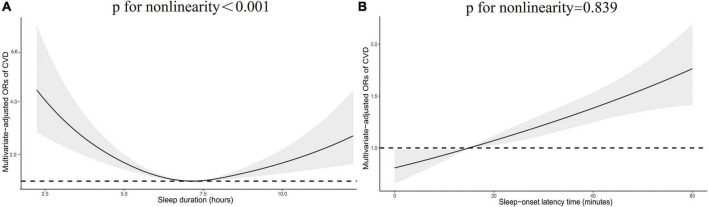
Restricted cubic spline plot of the association between total CVD and sleep duration **(A)** and sleep-onset latency time **(B)**. Adjustments were made according to age, sex, race, education, smoking status, alcohol consumption status, PIR, BMI, diabetes, hypertension, physical activity, TC, HDL-C, and TG. p indicates the results of a test for non-linearity. CVD, cardiovascular disease; OR, odds ratio.

Table 2 shows the association between sleep-related disorders and total CVD. In the fully adjusted model (Model 3), CVD was significantly associated with insufficient sleep (<7 h/night) as opposed to adequate sleep (7–8 h/night) (OR: 1.42; 95% CI 1.13, 1.78). Analyses of sleep-onset latency found that prolonged sleep-onset latency (>30 min/night) to fall asleep was associated with an increased prevalence of CVD (OR: 1.59; 95% CI 1.17, 2.15). In addition, the prevalence of CVD was 1.75 and 1.54 times higher among those who reported sleep problems and daytime sleepiness (OR: 1.75; 95% CI 1.41, 2.16 and OR: 1.54; 95% CI 1.25, 1.89). A higher prevalence of CVD was associated with excessive sleep (>9 h/night) and OSA symptoms in Model 1 (OR: 1.54; 95% CI 1.03, 2.31 and OR: 1.32; 95% CI 1.08, 1.61); however, the association disappeared in Model 2. In addition, we did not observe significant associations between short sleep-onset latency (<5 min/night) and CVD outcomes. Additionally, restricted cubic spline analysis confirmed a linear relationship between sleep-onset latency time and CVD (p for non-linearity = 0.839) and a non-linear relationship between sleep duration and CVD (p for non-linearity <0.001) (Figure 2).

**TABLE 2:** Adjusted odds ratios for associations between sleep-related disorders and total CVD.

	Model 1 OR (95% CI) *P*	Model 2 OR (95% CI) *P*	Model 3 OR (95% CI) *P*
**Sleep duration**			
<7 vs. 7–8 h	1.57 (1.26, 1.97) ***P < 0.001	1.44 (1.16, 1.80) **P = 0.008	1.42 (1.13, 1.78) *P = 0.025
>8 vs. 7–8 h	1.54 (1.03, 2.31) *P = 0.047	1.51 (0.97, 2.33) *P* = 0.092	1.43 (0.92, 2.22) *P* = 0.163
**Sleep-onset latency time**			
<5 vs. 5–30 min	0.79 (0.59, 1.06) *P* = 0.130	0.77 (0.58, 1.03) *P* = 0.108	0.77 (0.57, 1.02) *P* = 0.121
>30 vs. 5–30 min	1.77 (1.35, 2.32) ***P < 0.001	1.57 (1.17, 2.11) *P = 0.012	1.59 (1.17, 2.15) *P = 0.025
**Sleep problems**			
No	Reference	Reference	Reference
Yes	1.96 (1.62, 2.38) ***P < 0.001	1.74 (1.42, 2.13) ***P < 0.001	1.75 (1.41, 2.16) **P = 0.001
**OSA symptoms**			
No	Reference	Reference	Reference
Yes	1.32 (1.08, 1.61) *P = 0.011	1.13 (0.91, 1.40) *P* = 0.303	1.12 (0.89, 1.40) *P* = 0.367
**Daytime sleepiness**			
No	Reference	Reference	Reference
Yes	1.75 (1.44, 2.13) ***P < 0.001	1.52 (1.25, 1.85) **P = 0.001	1.54 (1.25, 1.89) **P = 0.004

OR, odds ratio; CI, confidence interval. Model 1 was adjusted for sex, age, and race. Model 2 was adjusted for sex, age, race, education, smoking status, alcohol consumption status, PIR, BMI, diabetes, and hypertension. Model 3 was adjusted for sex, age, race, education, smoking status, alcohol consumption status, PIR, BMI, diabetes, hypertension, activity, TC, HDL-C, and TG. The results are weighted based on the survey. *P < 0.05; **P < 0.01; ***P < 0.001.

### Association between sleep-related disorders and individual cardiovascular diseases

The association between sleep-related disorders and individual CVDs (CHF, CHD, AP, heart attack and stroke) was further analyzed by multivariate logistic regression (Table 3). In Model 3, sleep problems were associated with a high prevalence of CHF (OR: 2.28; 95% CI 1.69, 3.09), CHD (OR: 1.44; 95% CI 1.12, 1.85), AP (OR: 1.96; 95% CI 1.40, 2.74), heart attack (OR: 2.05; 95% CI 1.67, 2.53) and stroke (OR: 1.78; 95% CI 1.32, 2.40). Daytime sleepiness also showed a strong association with CHF (OR: 1.73; 95% CI 1.22, 2.46), AP (OR: 1.53; 95% CI 1.12, 2.10), heart attack (OR: 1.51; 95% CI 1.18, 1.95) and stroke (OR: 1.60; 95% CI 1.09, 2.36) in multiple logistic regression using the fully adjusted model (Model 3), but it was not associated with CHD. Furthermore, participants with insufficient sleep (<7 h/night) and prolonged sleep-onset latency (>30 min/night) also exhibited statistically significant associations with heart attack (OR: 1.59; 95% CI 1.19, 2.13 and OR: 1.76; 95% CI 1.29, 2.41) in Model 3. Moreover, according to Model 3, prolonged sleep-onset latency (>30 min/night) was associated with a 108% increased risk of CHF (OR: 2.08; 95% CI 1.33, 3.23), and short sleep-onset latency (<5 min/night) was associated with a 36% reduction in the risk of stroke (OR: 0.64; 95% CI 0.45, 0.90). Model 2, which was not adjusted for lipid levels or physical activity, showed that insufficient sleep (<7 h/night) (OR: 1.55; 95% CI 1.05, 2.29) and OSA symptoms (OR: 1.48; 95% CI 1.07, 2.05) were positively associated with AP outcomes. In addition, Model 2 revealed a correlation between prolonged sleep-onset latency (>30 min/night) and stroke (OR: 1.50; 95% CI 1.08, 2.08).

**TABLE 3:** Adjusted odds ratios for associations between sleep-related disorders and individual CVDs.

	Sleep duration OR (95% CI) *P*		Sleep-onset latency time OR (95% CI) *P*		Sleep problems OR (95% CI) *P*	OSA symptoms OR (95% CI) *P*	Daytime sleepiness OR (95% CI) *P*
	<7 vs. 7–8 h	>8 vs. 7–8 h	<5 vs. 5–30 min	>30 vs. 5–30 min			
**CHF**							
Model 1	1.46 (1.07, 1.99) *P = 0.026	1.58 (0.85, 2.95) *P* = 0.164	0.91 (0.61, 1.35) *P* = 0.652	2.34 (1.57, 3.48) ***P < 0.001	2.57 (1.93, 3.42) ***P < 0.001	1.49 (1.13, 1.97) *P = 0.010	1.93 (1.39, 2.69) ***P < 0.001
Model 2	1.33 (0.98, 1.79) *P* = 0.090	1.55 (0.82, 2.90) *P* = 0.201	0.87 (0.58, 1.30) *P* = 0.512	2.09 (1.35, 3.22) **P = 0.007	2.29 (1.70, 3.09) ***P < 0.001	1.24 (0.91, 1.68) *P* = 0.191	1.71 (1.22, 2.40) **P = 0.009
Model 3	1.31 (0.96, 1.78) *P* = 0.135	1.49 (0.79, 2.82) *P* = 0.269	0.87 (0.58, 1.30) *P* = 0.515	2.08 (1.33, 3.23) *P = 0.018	2.28 (1.69, 3.09) **P = 0.001	1.24 (0.93, 1.65) *P* = 0.188	1.73 (1.22, 2.46) *P = 0.018
**CHD**							
Model 1	1.21 (0.93, 1.57) *P* = 0.166	1.15 (0.68, 1.95) *P* = 0.613	0.89 (0.58, 1.35) *P* = 0.586	1.24 (0.81, 1.90) *P* = 0.325	1.58 (1.23, 2.04) **P = 0.002	1.38 (0.97, 1.96) *P* = 0.086	1.26 (0.96, 1.65) *P* = 0.108
Model 2	1.13 (0.89, 1.45) *P* = 0.343	1.17 (0.67, 2.02) *P* = 0.595	0.85 (0.55, 1.29) *P* = 0.460	1.15 (0.76, 1.74) *P* = 0.520	1.43 (1.12, 1.83) *P = 0.015	1.16 (0.81, 1.65) *P* = 0.431	1.16 (0.87, 1.55) *P* = 0.326
Model 3	1.10 (0.86, 1.41) *P* = 0.478	1.07 (0.61, 1.88) *P* = 0.812	0.83 (0.55, 1.26) *P* = 0.422	1.13 (0.76, 1.66) *P* = 0.568	1.44 (1.12, 1.85) *P = 0.026	1.14 (0.81, 1.62) *P* = 0.474	1.19 (0.88, 1.62) *P* = 0.289
**AP**							
Model 1	1.69 (1.15, 2.47) *P = 0.013	1.48 (0.74, 2.98) *P* = 0.280	0.87 (0.58, 1.30) *P* = 0.510	1.32 (0.77, 2.27) *P* = 0.325	2.17 (1.58, 3.00) ***P < 0.001	1.74 (1.27, 2.38) **P = 0.002	1.67 (1.24, 2.26) **P = 0.003
Model 2	1.55 (1.05, 2.29) *P = 0.049	1.48 (0.71, 3.10) *P* = 0.320	0.86 (0.58, 1.27) *P* = 0.461	1.21 (0.70, 2.09) *P* = 0.515	1.95 (1.42, 2.67) **P = 0.001	1.48 (1.07, 2.05) *P = 0.035	1.48 (1.10, 1.98) *P = 0.023
Model 3	1.56 (0.95, 2.56) *P* = 0.072	1.42 (0.68, 2.96) *P* = 0.389	0.84 (0.57, 1.24) *P* = 0.417	1.23 (0.71, 2.13) *P* = 0.485	1.96 (1.40, 2.74) **P = 0.005	1.46 (0.99, 2.16) *P* = 0.056	1.53 (1.12, 2.10) *P = 0.032
**Heart attack**							
Model 1	1.76 (1.33, 2.32) ***P < 0.001	1.46 (0.92, 2.32) *P* = 0.119	1.13 (0.81, 1.58) *P* = 0.494	1.98 (1.46, 2.68) ***P < 0.001	2.28 (1.84, 2.83) ***P < 0.001	1.25 (0.87, 1.78) *P* = 0.242	1.68 (1.29, 2.20) ***P < 0.001
Model 2	1.61 (1.22, 2.13) **P = 0.006	1.43 (0.87, 2.34) *P* = 0.186	1.13 (0.81, 1.57) *P* = 0.503	1.71 (1.25, 2.35) **P = 0.007	2.02 (1.65, 2.48) ***P < 0.001	1.09 (0.74, 1.60) *P* = 0.672	1.48 (1.14, 1.91) *P = 0.011
Model 3	1.59 (1.19, 2.13) *P = 0.020	1.33 (0.80, 2.22) *P* = 0.309	1.12 (0.80, 1.57) *P* = 0.542	1.76 (1.29, 2.41) *P = 0.012	2.05 (1.67, 2.53) ***P < 0.001	1.07 (0.73, 1.58) *P* = 0.739	1.51 (1.18, 1.95) *P = 0.015
**Stroke**							
Model 1	1.50 (1.00, 2.26) *P* = 0.050	1.56 (0.94, 2.58) *P* = 0.098	0.64 (0.46, 0.89) *P = 0.014	1.69 (1.23, 2.33) **P = 0.004	2.06 (1.53, 2.76) ***P < 0.001	1.33 (0.99, 1.78) *P* = 0.058	1.94 (1.34, 2.81) **P = 0.002
Model 2	1.38 (0.95, 2.02) *P* = 0.123	1.51 (0.91, 2.51) *P* = 0.137	0.62 (0.44, 0.88) *P = 0.020	1.50 (1.08, 2.08) *P = 0.034	1.80 (1.33, 2.43) **P = 0.002	1.15 (0.85, 1.55) *P* = 0.395	1.69 (1.15, 2.48) *P = 0.021
Model 3	1.31 (0.89, 1.91) *P* = 0.218	1.41 (0.85, 2.35) *P* = 0.236	0.64 (0.45, 0.90) *P = 0.045	1.39 (0.92, 2.09) *P* = 0.097	1.78 (1.32, 2.40) **P = 0.007	1.13 (0.83, 1.53) *P* = 0.465	1.60 (1.09, 2.36) *P = 0.048

OR, odds ratio; CI, confidence interval. Model 1 was adjusted for sex, age, and race. Model 2 was adjusted for sex, age, race, education, smoking status, alcohol consumption status, PIR, BMI, diabetes, and hypertension. Model 3 was adjusted for sex, age, race, education, smoking status, alcohol consumption status, PIR, BMI, diabetes, hypertension, activity, TC, HDL-C, and TG. The results are weighted based on the survey. *P < 0.05; **P < 0.01; ***P < 0.001. CHF, congestive heart failure; CHD, coronary heart disease; AP, angina pectoris.

### Subgroup analyses between sleep-related disorders and total cardiovascular disease

The results of stratified analyses by different variables, including age, sex, obesity, abdominal obesity, diabetes, hypertension, and physical activity, are presented in Tables 4, 5. Analysis confirmed that the prevalence of CVD was positively correlated with prolonged sleep-onset latency, sleep problems and daytime sleepiness. There was no significant trend of association between CVD and prolonged sleep-onset latency in females, participants over 60 years of age, and participants with obesity, abdominal obesity, hypertension, diabetes and physical inactivity. In addition, there was no significant trend toward an association between CVD and sleep problems in participants without hypertension or abdominal obesity. Subgroup analysis stratified by age, abdominal obesity, diabetes, and physical activity suggested that insufficient sleep is associated with CVD. Subgroup analysis stratified by sex and physical activity confirmed that CVD was inversely correlated with short sleep-onset latency. No interactions were found except in the analyses of the association of sleep problems with total CVD stratified by age (p for interaction = 0.019) and the association of short sleep-onset latency with total CVD stratified by sex (p for interaction = 0.049).

**TABLE 4:** Subgroup analysis of the associations of sleep duration and sleep-onset latency time with total CVD.

	Sleep duration OR (95% CI) *P*				Sleep-onset latency time OR (95% CI) *P*			
	<7 vs. 7–8 h	*P* for interaction	>8 vs. 7–8 h	*P* for interaction	<5 vs. 5–30 min	*P* for interaction	>30 vs. 5–30 min	*P* for interaction
**Age**		0.441		0.379		0.455		0.170
<60 year	1.56 (1.09, 2.22) *P = 0.044		1.02 (0.50, 2.11) *P* = 0.949		0.68 (0.38, 1.21) *P* = 0.229		1.77 (1.21, 2.60) *P = 0.022	
≥60 year	1.38 (0.93, 2.04) *P* = 0.095		1.70 (0.92, 3.14) *P* = 0.080		0.88 (0.60, 1.29) *P* = 0.524		1.28 (0.83, 1.97) *P* = 0.295	
**Gender**		0.249		0.432		*0.049		0.578
Male	1.29 (0.99, 1.68) *P* = 0.107		1.21 (0.80, 1.85) *P* = 0.397		0.98 (0.69, 1.41) *P* = 0.926		1.87 (1.30, 2.68) *P = 0.011	
Female	1.55 (0.98, 2.45) *P* = 0.057		1.64 (0.92, 2.94) *P* = 0.139		0.52 (0.33, 0.81) *P = 0.023		1.33 (0.89, 2.00) *P* = 0.207	
**Obesity**		0.932		0.787		0.725		0.487
BMI ≥ 30	1.35 (0.97, 1.88) *P* = 0.116		1.43 (0.80, 2.56) *P* = 0.263		0.81 (0.55, 1.18) *P* = 0.304		1.37 (0.85, 2.20) *P* = 0.234	
BMI < 30	1.46 (0.93, 2.28) *P* = 0.086		1.46 (0.84, 2.55) *P* = 0.220		0.73 (0.51, 1.06) *P* = 0.139		1.73 (1.27, 2.37) *P = 0.011	
**Abdominal obesity**		0.252		0.381		0.660		0.454
Yes	1.52 (1.19, 1.95) *P = 0.016		1.65 (0.92, 2.95) *P* = 0.079		0.74 (0.53, 1.03) *P* = 0.128		1.46 (0.98, 2.18) *P* = 0.114	
No	1.17 (0.75, 1.84) *P* = 0.508		1.04 (0.54, 2.01) *P* = 0.913		0.83 (0.56, 1.24) *P* = 0.398		1.98 (1.32, 2.99) *P = 0.014	
**Diabetes**		0.455		0.695		0.537		0.745
Yes	1.13 (0.74, 1.73) *P* = 0.580		1.27 (0.63, 2.56) *P* = 0.523		0.90 (0.56, 1.44) *P* = 0.665		1.41 (0.73, 2.73) *P* = 0.337	
No	1.51 (1.13, 2.01) *P = 0.025		1.44 (0.84, 2.45) *P* = 0.220		0.73 (0.51, 1.05) *P* = 0.127		1.62 (1.22, 2.16) *P = 0.010	
**Hypertension**		0.574		0.699		0.210		0.363
Yes	1.33 (0.96, 1.85) *P* = 0.074		1.47 (0.93, 2.34) *P* = 0.147		0.84 (0.62, 1.15) *P* = 0.316		1.42 (0.98, 2.06) *P* = 0.105	
No	1.64 (0.93, 2.90) *P* = 0.080		1.25 (0.64, 2.47) *P* = 0.536		0.57 (0.32, 1.03) *P* = 0.060		2.33 (1.45, 3.74) *P = 0.010	
**Physical activity**		0.509		0.139		0.277		0.436
Active	1.43 (0.98, 2.08) *P* = 0.060		0.86 (0.42, 1.77) *P* = 0.695		0.80 (0.52, 1.21) *P* = 0.320		1.99 (1.35, 2.93) **P = 0.008	
Inactive	1.48 (1.08, 2.02) *P = 0.042		1.95 (1.15, 3.31) *P = 0.038		0.70 (0.53, 0.93) *P = 0.038		1.31 (0.90, 1.89) *P* = 0.192	

OR, odds ratio; CI, confidence interval. Analyses were adjusted for sex, age, race, education, smoking status, alcohol consumption status, PIR, BMI, diabetes, hypertension, activity, TC, HDL-C, and TG when they were not the strata variables. The results are weighted based on the survey. *P < 0.05; **P < 0.01.

**TABLE 5:** Subgroup analysis of the associations of sleep-related disorders with total CVD.

	Sleep problems OR (95% CI) *P*	*P* for interaction	OSA symptoms OR (95% CI) *P*	*P* for interaction	Daytime sleepiness OR (95 %CI) *P*	*P* for interaction
**Age**		*0.019		0.361		0.273
<60 year	2.74 (1.74, 4.32) **P = 0.003		1.19 (0.79, 1.79) *P* = 0.437		1.54 (1.09, 2.17) *P = 0.039	
≥60 year	1.29 (1.14, 1.68) *P = 0.037		1.04 (0.84, 1.28) *P* = 0.736		1.36 (1.14, 1.62) **P = 0.009	
**Gender**		0.841		0.519		0.351
Male	1.77 (1.38, 2.27) **P = 0.002		1.04 (0.77, 1.41) *P* = 0.808		1.19 (0.89, 1.60) *P* = 0.268	
Female	1.72 (1.21, 2.43) *P = 0.016		1.19 (0.84, 1.68) *P* = 0.364		1.85 (1.36, 2.53) **P = 0.004	
**Obesity**		0.692		0.997		0.226
BMI ≥ 30	1.82 (1.36, 2.43) **P = 0.004		1.10 (0.73, 1.66) *P* = 0.653		1.24 (0.91, 1.68) *P* = 0.203	
BMI < 30	1.63 (1.17, 2.26) *P = 0.020		1.13 (0.92, 1.40) *P* = 0.275		1.75 (1.29, 2.37) **P = 0.007	
**Abdominal obesity**		0.622		0.715		0.384
Yes	1.76 (1.40, 2.22) **P = 0.002		1.05 (0.76, 1.46) *P* = 0.769		1.41 (1.13, 1.77) *P = 0.020	
No	1.61 (0.97, 2.69) *P* = 0.063		1.30 (0.93, 1.82) *P* = 0.169		1.97 (1.23, 3.15) *P = 0.022	
**Diabetes**		0.801		0.373		0.899
Yes	1.67 (1.21, 2.32) *P = 0.015		0.97 (0.64, 1.45) *P* = 0.880		1.39 (0.97, 1.97) *P* = 0.107	
No	1.74 (1.34, 2.27) **P = 0.003		1.21 (0.95, 1.54) *P* = 0.150		1.53 (1.19, 1.98) **P = 0.009	
**Hypertension**		0.560		0.944		0.285
Yes	1.83 (1.41, 2.36) **P = 0.002		1.12 (0.88, 1.42) *P* = 0.391		1.42 (1.17, 1.73) **P = 0.008	
No	1.44 (0.87, 2.38) *P* = 0.189		1.04 (0.58, 1.87) *P* = 0.905		1.99 (1.25, 3.16) *P = 0.019	
**Physical activity**		0.230		0.341		0.771
Active	1.59 (1.08, 2.32) *P = 0.042		1.27 (0.87, 1.84) *P* = 0.250		1.67 (1.23, 2.26) **P = 0.009	
Inactive	1.93 (1.50, 2.48) ***P < 0.001		0.98 (0.78, 1.23) *P* = 0.859		1.46 (1.14, 1.87) *P = 0.016	

OR, odds ratio; CI, confidence interval. Analyses were adjusted for sex, age, race, education, smoking status, alcohol consumption status, PIR, BMI, diabetes, hypertension, activity, TC, HDL-C, and TG when they were not the strata variables. The results are weighted based on the survey. *P < 0.05; **P < 0.01; ***P < 0.001.

## Discussion

Epidemiological studies on the relationship between sleep-related disorders and the prevalence of total and specific CVDs in non-institutionalized adults (20 years and older) of the United States are scarce. Our study fully considered the sex, age, race/ethnicity, education, and socioeconomic status of the participants, and a broad range of confounders were controlled for. In this large, nationally representative study population, this study examined the relationship between sleep disorders and the prevalence of total and specific CVDs through epidemiological research. We found that insufficient sleep, prolonged sleep-onset latency, sleep problems and daytime sleepiness may be positively correlated with CVD. Furthermore, participants who reported sleep problems and daytime sleepiness were also generally associated with specific CVDs, with the exception of the association of daytime sleepiness with CHD. Participants who reported prolonged sleep-onset latency were also more likely to have CHF and heart attack, Additionally, shortened sleep-onset latency may be protective against stroke. The likelihood of heart attack also increased with insufficient sleep. In addition, we found a positive non-linear correlation between CVD prevalence and sleep duration and a positive linear correlation between CVD prevalence and sleep-onset latency time. Stratified analysis further indicated that the prevalence of CVD was positively correlated with prolonged sleep-onset latency, sleep problems and daytime sleepiness. It is worth mentioning that participants younger than 60 years and with sleep problems had a higher risk of CVD than those older than 60 years, and shortened sleep-onset latency may be a CVD protective factor in females.

To date, several studies have examined the associations between sleep-related disorders and CVD. A prospective study of 385,292 United Kingdom biobank participants demonstrated that healthy sleep patterns, including adequate sleep, no sleep problems, no snoring and no frequent excessive daytime sleepiness, are associated with a reduced risk of CVD, CHD, and stroke among participants with low, intermediate, or high genetic risk (27). Another prospective study of the United Kingdom biobank suggests that lifestyle-related CVD risk is modified by sleep patterns; among participants with a poor sleep pattern, an unfavorable lifestyle (per score increase) was associated with a 25% increased risk for CVD (28). The conclusions of these two studies are similar to our study, but their research focuses on exploring healthy combined sleep patterns to avoid CVD risk. It is worth mentioning that our research subdivided sleep-related disorders and linked them one by one with total and specific CVDs to find more specific correlations. In addition, this is a cross-sectional study, and the results should be interpreted with caution.

Many longitudinal studies have confirmed that insufficient sleep is associated with an increased risk of many CVDs. A meta-analysis of prospective cohort studies suggested that U-shaped associations were indicated between sleep duration and the risk of total CVD, CHD, and stroke, and insufficient sleep or excessive sleep increases the risk of all outcomes (10, 11). Previous Mendelian randomization studies conducted by Ai et al have also suggested causal adverse effects of genetically predicted short sleep duration on a broad range of CVDs, including myocardial infarction and coronary artery disease (29). In our study, we only observed an association of insufficient sleep with total CVD and heart attack, and the reason for speculation is that the conclusions of our study come from a cross-sectional study of healthy participants. Controversy exists regarding the effects of excessive sleep and CVD. Although some evidence suggests that excessive sleep may be more detrimental to cardiovascular health than insufficient sleep (30), it is unknown whether excessive sleep directly increases the risk of CVD or indirectly through other factors. To the best of our knowledge, excessive sleep is associated with a number of psychiatric diseases and their drug use and unhealthy lifestyles (31–33), such as depression, benzodiazepine use, obesity, and sedentary lifestyles. Currently, our study provides epidemiological evidence of no statistically significant association between excessive sleep and the prevalence of total and specific CVDs. In addition, the results predicted from genetic susceptibility are also consistent with our conclusions (29).

Our study defines sleep problems as a large collection of sleep disorders, including insomnia, poor sleep quality and difficulty falling asleep, which is in line with the definition of the American Academy of Sleep Medicine (34). While the pathogenesis of sleep-related disorders with CVD is not fully understood, there appear to be multiple pathophysiological mechanisms, with the prevailing view including dysregulation of the hypothalamic-pituitary axis (35, 36), abnormal modulation of the autonomic nervous system and increased sympathetic nervous system activity (3), and increased systemic inflammation (37, 38). There have already been prospective meta-analyses reporting insomnia with an increased risk of CVD, and they define insomnia symptoms almost identical to our definition of sleep problems (39, 40). It is worth mentioning that our study subdivided CVD and found that sleep problems were generally associated with total and specific CVDs. Additionally, the associations of sleep problems with CVD risk were more pronounced in the group younger than 60 years. The MONICA-brianza and PAMELA cohort studies found that the severe effect of sleep disorders on CVD began at age 48, suggesting that this association seems to be age-related (41). A Korean study failed to find any significant association between short sleep duration and hypertension in elderly subjects aged ≥65 years, and the authors explained that the elderly may compensate for shorter sleep duration at night by napping during the day (42). There is much evidence that the social stress faced by middle-aged people can significantly affect sleep quality and may increase the risk of CVD (43–45). Furthermore, the presence of sleep disorders marks an increase in CVD risk factors and is associated with health-related behavioral changes (46, 47). Another possibility is that bad habits affecting sleep quality in the middle-aged population, such as sedentary lifestyle, screen use time, and sleep deprivation during working hours, indirectly amplify the effect on CVD but are not significant in the elderly population.

In addition, prolonged sleep-onset latency may be indicative of having a sleep disorder and reduce sleep efficiency (21, 22). Our current study only found associations with total CVD, CHF, and heart attack and a statistical trend toward an association with stroke. Shorter sleep-onset latency can result in high quality sleep or excessive drowsiness compared to longer sleep-onset latency, Our study tends favor to the former and suggests that shorter sleep-onset latency may be a protective factor against stroke and that females are more likely to benefit from this effect *via* a reduced CVD risk. There are few relevant studies on the mechanism of sleep-onset latency, which still needs to be further explored. Previous studies have shown that OSA may be an independent risk factor for CVD and is associated with increased cardiovascular mortality (48, 49), but our results suggest no association, which is consistent with previous cross-sectional studies based on the NHANES (50). Therefore, the results should be interpreted. The reason for our analysis may be that polysomnographic information was not used to confirm the diagnosis of sleep apnea, thus deviating from the OSA definition, and self-reported sleep information is associated with recall bias.

Our study has several important advantages over previous studies. The results of a large, nationwide, randomized sample survey can be generalized to the adult non-institutionalized population in the United States. Associations between multiple sleep disorders and the prevalence of total and specific CVDs were analyzed separately, thus improving the comprehensiveness and accuracy of the analysis. However, several limitations of this study warrant attention. The cross-sectional nature of this study precludes the determination of causality. Self-reported sleep symptoms can also be considered a limitation with possible recall bias without further objective measures or clinical assessment. Despite potential differences between subjective and objective sleep assessments, both are clinically important (51). While we adjusted for confounders, the lack of important variables regarding sleep and CVD, such as hypnotic use, caffeine intake, and presence of depression, should also be considered a study limitation. Moreover, we did not exclude special populations such as cancer and pregnancy. The exclusion of patients due to missing measurement data may have biased the results.

## Conclusion

This current national population-based study suggests that the prevalence of total CVD was possibly associated with insufficient sleep, prolonged sleep-onset latency, sleep problems and daytime sleepiness. Furthermore, we found a positive non-linear correlation between CVD prevalence and sleep duration and a positive linear correlation between CVD prevalence and sleep-onset latency time. Individual CVDs could be associated with certain sleep-related disorders. In addition, our study also makes a unique contribution indicating that the risk of CVD in participants younger than 60 years and with sleep problems should be considered. Furthermore, shortened sleep-onset latency may be a CVD protective factor in females. Sleep is associated with lifelong health status, and healthy sleep should be as important as avoiding other risk factors in promoting overall cardiovascular health. Future large prospective studies are also needed to validate our conclusions.

## Data availability statement

The datasets presented in this study can be found in online repositories. The names of the repository/repositories and accession number(s) can be found below: https://wwwn.cdc.gov/nchs/nhanes/.

## Ethics statement

The studies involving human participants were reviewed and approved by the NCHS Research Ethics Review Committee. The patients/participants provided their written informed consent to participate in this study.

## Author contributions

KK and LQ contributed to the conception and design, acquisition, analysis, interpretation of the data, and drafting of the manuscript or critical revision for important intellectual content. AA, RR, DD, Y-YD, and HM collected and organized data. XM and Y-TM contributed to the conception and design and reviewing of the manuscript or critical revision for important intellectual content. All authors approved the final version, and agree to be accountable for all aspects of the work.
